# Study on the Changes of Bone Calcium during the Fermentation of Bone Powders with Different Fermenters

**DOI:** 10.3390/foods13020227

**Published:** 2024-01-11

**Authors:** Jia Meng, Ying Wang, Jinxuan Cao, Wendi Teng, Jinpeng Wang, Yuemei Zhang

**Affiliations:** 1 Key Laboratory of Geriatric Nutrition and Health, Beijing Technology and Business University (BTBU), Ministry of Education, Beijing 100048, Chinacaojinxuan@btbu.edu.cn (J.C.); jpwang@btbu.edu.cn (J.W.); zhangyuemei@btbu.edu.cn (Y.Z.); 2 Beijing Advanced Innovation Center for Food Nutrition and Human Health, Beijing Technology and Business University (BTBU), Beijing 100048, China; 3 School of Food and Health, Beijing Technology and Business University (BTBU), Beijing 100048, China

**Keywords:** bone powders, fermentation, calcium forms, calcium release, structure

## Abstract

Two fermenters, *Lactobacillus acidophilus* (LA) and the active dry yellow wine yeast (HY), were utilized to ferment cattle bones in order to release calcium. The influences of fermenters and the fermentation process on the calcium release capacity, particle properties, morphology, and chemical composition of bone powders were assessed, and the underlying mechanism was discussed. The results showed that LA had a better capacity of acid production than yeast, and therefore released more calcium during the fermentation of bone powders. The released calcium in the fermentation broth mainly existed in the forms of free Ca^2+^ ions, organic acid-bound calcium and a small amount of calcium–peptide chelate. For bone powders, the fermentation induced swollen bone particles, increased particle size, and significant changes of the internal chemical structure. Therefore, fermentation has a great potential in the processing of bone-derived products, particularly to provide new ideas for the development of calcium supplement products.

## 1. Introduction

As a critical mineral element in the human body, calcium accounts for about 1.5–2% of the human body’s weight, which mostly deposits in the form of phosphate in bones and teeth. The remaining calcium exists as free cations in soft tissues, cells, and blood, and plays a crucial role in numerous biochemical processes such as cell signaling, blood pressure control, hormone secretion, blood coagulation, etc. [1]. Calcium deficiency can cause not only osteoporosis, but also hypertension, kidney stones, and colon cancer [2]. The dietary reference values of calcium have been established in the need of health maintenance, which range from 1000 mg to 1300 mg depending on the reference guidelines [3]. However, limited calcium-rich foods and unbalanced dietary habits have hindered people to take sufficient dietary calcium, arousing the demand for calcium supplementation. Various calcium supplements have been developed, such as calcium salts, organic calcium salts, amino acid chelated calcium, and peptide calcium chelators, which provide satisfactory calcium content and are acknowledged as sources of daily calcium intake [4]. Biocalcium derived from byproducts of food processing has attracted great interest due to the low cost, bioavailability, good stability, and easy absorption in the gastrointestinal tract; for example, biocalcium from egg shell has been reported to supply a high proportion of soluble calcium salts [5,6,7,8].

Increasing concerns regarding biocalcium sources have been raised recently. Apart from egg shell [9], animal bone, a unique biocomposite containing approximately 65–70% hydroxyapatite (HAP, Ca_10_(PO_4_)_6_(OH)_2_) and 30–35% organics on a dry basis, has been recognized as a natural, low-cost source of calcium due to the good bioavailability and biocompatibility [10]. However, most bones are discarded in the slaughterhouse or used in feed production with low value, causing an enormous waste of resources, environmental pollution, and low economic benefits. The conversion of animal bones into calcium supplements not only mitigates environmental pollution, but also enhances the value of byproducts, thereby augmenting economic returns. It has been recognized that animal bones possess hierarchical structures with hydroxyapatite crystals embedded in the collagen fibril matrix. The utilization of biocalcium from bones is facing great challenges due to the complex structure of bones and the stability of HAP compounds.

Various efforts have been exerted in the extraction of biocalcium from animal bones. For example, fish bone was calcined at high temperature in order to extract HAP compounds [11]. Dynamic high-pressure microfluidization treatment showed an apparent capability to increase the efficiency of Ca^2+^ release by decreasing the particle size and changing the surface composition [12]. Thermal treatment is another effective method to enhance the release of calcium from fish bone through the degradation of the collagen fiber matrix and reducing the mechanical strength of the bone [13]. Nevertheless, the calcium extracted in these reports was in the form of inorganic calcium or ionized calcium, which has low adsorption and easy precipitation [14]. To overcome these shortcomings, enzymatic hydrolysis and fermentation have been applied. Wang et al. prepared peptide–calcium chelates by a combination of enzymatic hydrolysis and lactic acid bacteria fermentation [15]. Xu et al. produced calcium lactate by *B. coagulans* H-1 fermentation [16], with efficient calcium lactate production by fermentation coupled with crystallization-based in situ product removal. Calcium and bioactive peptides have been obtained from chicken bone and meat residues to produce peptide–calcium chelates, which enhanced the absorption of calcium relative to CaCl_2_ [9]. The fermentation of animal bones can yield valuable biocalcium, aligning with the principles of a circular economy and resource efficiency. Fermentation can control the growth and reproduction of some pathogens in food, which can improve food safety [17]. Through fermentation technology, calcium from bone waste is released and used to prepare calcium supplements, which can reduce the waste of bone resources and environmental pollution problems. Nevertheless, there is a paucity of literature addressing the impact of various fermenters on biocalcium release and the characterization of calcium species. Additionally, the underlying mechanism governing the release of calcium from bone powders remains elusive.

Our work aimed to investigate the mechanism of calcium release and the transformation of calcium forms during the fermentation of cattle bone powders by Lactobacillus acidophilus and yeast. Correspondingly, the physiochemical properties of cattle bone powders and the broth after different fermentation treatments were investigated and compared, including the morphology, structure, calcium release properties, and calcium species. These results could provide the theoretical basis for exploring natural fermented calcium-rich products, as well as improving the utilization and financial benefits of cattle bone.

## 2. Materials and Methods

### 2.1. Materials

Cattle bone powders were purchased from Henan PROTIL Food Technology Co., Ltd. (Hebi, China). *Lactobacillus acidophilus* (HH-LA26) powder was purchased from Xian Michel Biotechnology Co., Ltd. (Xian, China). The active dry yellow wine yeast (*Saccharomyces cerevisiae*) was purchased from Angel Yeast Co., Ltd. (Yichang, China). D-(+)-Glucose monohydrate was purchased from Shanghai Macklin Biochemical Co., Ltd. (Shanghai, China). Peptone was purchased from Beijing Solarbio Science & Technology Co., Ltd. (Beijing, China). Sodium hydroxide standard and sodium metaphosphate were purchased from Mreda Technology Co., Ltd. (Beijing, China).

### 2.2. Preparation of Samples

Preparation of the cattle bone powders medium: 2 wt% bone powders, 82 wt% water, 10 wt% dextrose, and 5 wt% peptone was mixed, pH adjusted to 6.5, and sterilized (at 121 °C for 20 min) in a vertical automatic pressure steam sterilizer (GI54TW, Zealway Instrument Co., Ltd., Xiamen, China), and then cooled down to room temperature. “wt%” is the weight content percentage (%).

Preparation of fermentation broth of cattle bone powders: 1 wt% *Lactobacillus acidophilus* powders or 1 wt% active dry yellow wine yeast was added to the medium, respectively, under aseptic conditions, which was followed by fermentation for 72 h in a full-temperature oscillating incubator (MQR-S1R, Shanghai Minquan Instruments Co., Ltd., Shanghai, China). The fermentation temperatures for *Lactobacillus acidophilus* and yeast were set at 37 °C and 30 °C, respectively, under anaerobic conditions. Blank group without fermenter was prepared as the Control group, which is denoted as C. All treatments were performed in triplicate.

Preparation of fermented cattle bone powders: After cooling down to room temperature (25 °C), the broth was centrifuged at 300× *g* for 5 min. The obtained precipitate was collected, washed three times repeatedly with deionized water, and dried at 50 °C to obtain fermented bone powder samples.

The fermentation broth produced by Lactobacillus acidophilus and active dry yellow wine yeast were denoted as LA and HY, whereas the fermented bone powders were denoted as LAB and HYB respectively. Bone powders in the Control group were referred as CB.

### 2.3. Determination of pH and Total Acid in Fermentation Broth

The pH value was determined using a pH meter (FE28, Mettler-Toledo International Inc., Zurich, Switzerland). Total acid in fermentation broth was determined by titration using a pH meter. Approximately 25 mL of the fermentation broth sample was titrated with 0.1 mol/L sodium hydroxide standard titration solution until the pH value reached 8.2, and the consumed volume of sodium hydroxide solution was recorded. The total acid content was calculated based on the following equation:

(1) $$X=\frac{\left[C\times \left(V_{1}-V_{2}\right)\right]\times k\times F}{m}\times 1000$$

where *X* is the total acid content of the sample, g/L; *C* is the concentration of a standard titration solution of sodium hydroxide, mol/L; *V*_1_ is the volume of standard titration solution of sodium hydroxide consumed in the titration of the sample solution, mL; *V*_2_ is the volume of standard titration solution of sodium hydroxide consumed in the blank test, mL; *k* is the conversion factor of 0.090 for lactic acid [18]; *F* is the dilution times of the sample; and *m* is the volume of the aspirated sample, mL.

### 2.4. Determination of Morphological Distribution and Content of Calcium in Fermentation Broths

The bone powder fermentation broth was centrifuged at 300× *g* for 10 min at 4 °C, and the supernatant was collected to measure the total calcium content by inductively coupled plasma atomic emission spectrometer (ICPOES730, Agilent Technologies, Santa Clara, CA, USA). The total calcium content in the fermentation broth is recorded as *ω* mg/L. Then, 5 mL of fermentation broth was taken in a centrifuge tube and 25 mL of anhydrous ethanol was added to precipitate the protein and peptide. After centrifugation at 10,000 r/min for 10 min, the supernatant and precipitate were obtained. The precipitate was dried at 35 °C and the calcium content in the precipitate was determined using ICP (inductively coupled plasma), which was denoted as protein/peptide-bound calcium *ω*_1_ mg/L. Free calcium content in the supernatant was determined by calcium ion selective electrode (PXSJ-216F, Shanghai INESA Scientific Instruments Co., Ltd., Shanghai, China), and the amount of free calcium ions is recorded as *ω*_2_ mg/L. The content of organic acid complexed calcium in the fermentation broth is recorded as *ω*_3_ and calculated based on the equation of *ω*_3_ = *ω − ω*_1_ *− ω*_2_.

### 2.5. Measurement of Particle Size

The mean particle size of cattle bone powders was assayed by a laser diffraction particle size analyzer (SALD-3000, Shimadzu Co., Kyoto, Japan). Before testing, the bone powder samples were made into a suspension of 1 mg/g with 0.2% sodium hexametaphosphate solution and dispersed by ultrasonication for 15 min.

### 2.6. Microstructure of Bone Powders

The microstructure of the fermented bone powders was analyzed by a scanning electron microscope (JEOL JSM-6390LV, Tokyo, Japan) at an acceleration voltage of 25 kV, with the powder samples sprayed and sputter-coated with gold (Sputter coater SPI-Module, West Chester, PA, USA).

### 2.7. Specific Surface Area and Pore Size Analysis of Bone Powders

Determination of specific surface area and pore size of fermented bone powders was performed using a surface area and porosimetry analyzer (V-Sorb 2800, Gold APP Instruments Corporation, Beijing, China). The samples were dried at 100 °C and then put into sample tubes to be degassed in a vacuum environment for 4 h. Nitrogen adsorption experiments were carried out at −196 °C and relative pressures of 0–1 MPa. The specific surface area, pore volume, and pore size were determined by the multipoint Brunauer–Emmett–Teller (BET) adsorption characterization, and the pore size of the samples was calculated by the Barret–Joyner–Halenda (BJH) method.

### 2.8. X-ray Diffraction

The X-ray diffraction (XRD) analyses were carried out using a diffractometer (D8 Advance, Bruker Technologies GmbH, Rheinstetten, Germany) to investigate the phase composition and crystal structure of the bone powders and calcium compounds in fermentation broth. The diffractometer was operated at 40 kV and 40 mA, and patterns were acquired in the 2θ range from 10° to 90° at a scanning speed of 6°/min.

### 2.9. Fourier-Transform Infrared Spectroscopy 

Two milligram samples (the fermented bone powders or calcium compounds in fermentation broth) were mixed with 100 mg dry KBr and pressed into pellets. The FT-IR spectra were recorded using an infrared spectrophotometer (Nicolet 6700, Thermo-Nicolet Co., Madison, WI, USA) in the range of 4000–400 cm^−1^ at a resolution of 4 cm^−1^. The peak signals in the spectra were analyzed by Omnic software (Version 7.3, Thermo Nicolet Co., Madison, WI, USA).

### 2.10. Determination of Calcium–Phosphorus Ratio in Bone Powders

The contents of calcium and phosphorus in bone powders were measured using ICP, on which, the calcium–phosphorus ratio was calculated.

### 2.11. Thermogravimetric Analysis

Thermogravimetric analysis of fermented bone powders and calcium compounds in fermentation broth were determined with a thermal analyzer (STA 2500 Regulus, Netzsch Geraetebau GmbH, Selb, Germany). The sample was placed in an Al_2_O_3_ crucible and heated at a constant rate of 10 °C /min in the temperature range of 30 to 900 °C under an airflow.

### 2.12. Statistical Analysis

We conducted all measurements in triplicate, and all data are shown as mean ± standard deviation of the mean. The statistical analyses were conducted with SPSS version 26 (SPSS Inc., Chicago, IL, USA). Statistical significance was established by one-way analysis of variance (ANOVA) followed by Duncan’s multiple range test, and means were deemed to be differentially significant at *p* < 0.05.

## 3. Results

### 3.1. Effects of Fermentation on pH and Total Acid Content in Bone Powder Fermentation Broths

**Figure 1:**
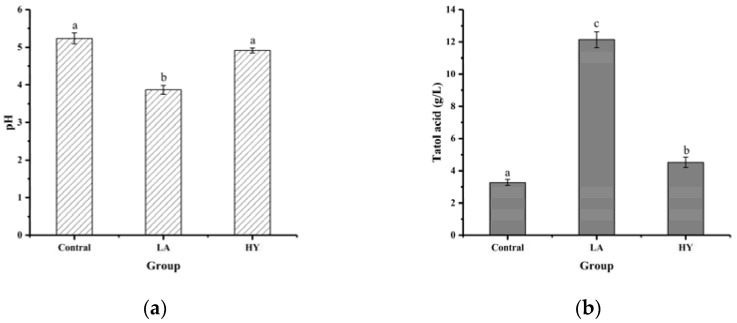
Comparison of pH (**a**) and total acid content (**b**) of different bone powder fermentation broths. The different letters indicate that differences are significant (*p* < 0.05).

The results of pH and total acid content in bone powder fermentation broths are shown in Figure 1. As depicted in Figure 1a, after 72 h of fermentation, the pH values of fermentation broths in LA and HY groups were reduced from 5.23 in Control group to 3.87 and 4.92, respectively. The total acid content of the LA and HY groups in Figure 1b was 12.138 g/L and 4.519 g/L, respectively, 3.71 and 1.38 times more than the Control group. Compared with the Control group, the decrease of pH values and the increase of total acid content in the LA and HY groups indicated the production of acids in the growth of Lactobacillus acidophilus and active dry yellow wine yeast. The total acid content of the LA group was obviously higher than the HY group, revealing that Lactobacillus acidophilus had a better growth, reproduction ability, and acid production capacity in the medium than the active dry yellow wine yeast.

Bones were rich in bone minerals and collagen, which provided essential nutrients for the growth and reproduction of the strain. Both *Lactobacillus acidophilus* and yeast could metabolize glucose with the production of organic acids in the fermentation process under microaerophilic conditions, despite having different fermentation mechanisms. *Lactobacillus acidophilus* had been reported to produce 2 mol pyruvate/mol glucose, which was reduced to 2 mol lactic acid as an electron acceptor in the catalytic reaction of lactate by the Embden–Meyerhof (EM) pathway [19]. By contrast, glucose broke down into pyruvate with a small amount of ATP (adenosine triphosphate) and NADH (nicotinamide adenine dinucleotide) through the TCA (tricarboxylic acid) cycle in the cytoplasm of the yeast cells, which was followed by the decarboxylation of pyruvate, leading to the formation of acetaldehyde and release of carbon dioxide. Then, acetaldehyde reacted with NADH to produce ethanol and regenerate NAD^+^ under anoxic or slightly acidic conditions [20]. Therefore, LA exhibited a better capability of producing acid than yeast.

### 3.2. Analysis of Calcium Content and Morphology in the Fermentation Broths

**Figure 2:**
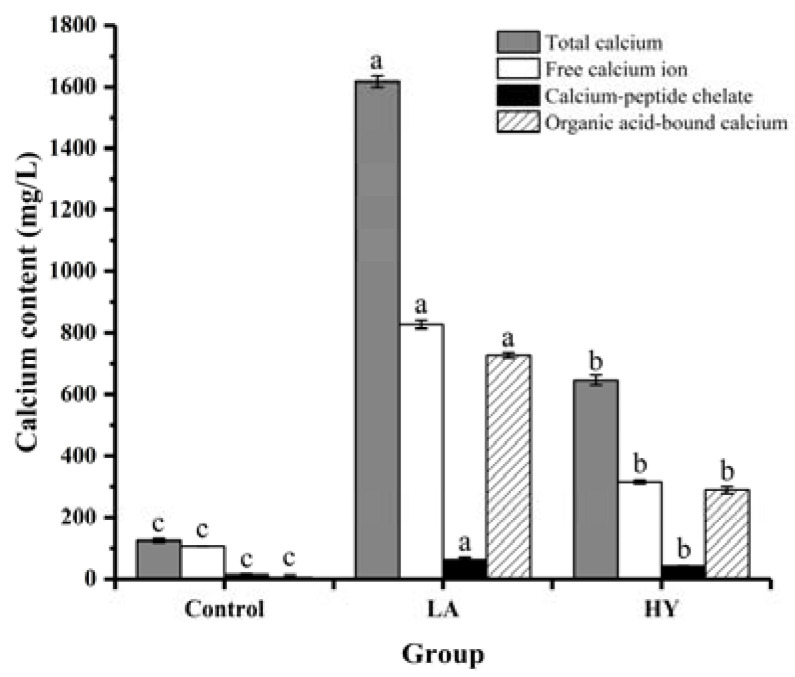
Calcium forms and content of fermentation broth by various fermentation agents. The different letters indicate that differences are significant (*p* < 0.05).

The forms and contents of calcium in the fermentation broth are shown in Figure 2 and Table 1. It was notable that significant differences existed in the contents of different forms of calcium in the fermentation broths (*p* < 0.05). At the end of fermentation, the total calcium contents of LA and HY groups were remarkably higher than that of Control group (124.90 mg/L), which were 646.57 mg/L and 1617.47 mg/L, respectively. Apparently, LA released the most content of calcium from the fermentation of bone powders, which might be attributed to the good acid-producing capacity. Bone is a structural biological composed of a mineral part (HAP, hydroxyapatite) and an organic part (collagen). HAP and collagen, as the fundamental building blocks of bone, are arrayed in different length scales to form a complicated hierarchical structure [21,22,23]. Organic acids destroyed the structure of the collagen molecule and the links between collagen and hydroxyapatite (HAP) (formed by collagen sharing hydroxyl groups), leading to the dissolution of collagen and HAP, and facilitating the release of Ca^2+^ [24]. Collagen was hydrolyzed by proteolytic enzymes produced during the fermentation process to produce collagen polypeptides [25,26]. Some free Ca^2+^ ions could be chelated with the organic acids that were produced during the fermentation process, leading to the formation of organic acid-bound calcium, whereas some calcium ions could be chelated by carboxyl oxygen and amino nitrogen atoms of collagen peptides to produce peptide–calcium chelate [27]. The experimental results showed that the calcium element in the fermentation broths was mainly in the form of free calcium, organic acid-bound calcium, and calcium–peptide chelate. It was apparent that the calcium content was in the following order: free calcium > organic acid-bound calcium > calcium–peptide chelate. The contents of free calcium, calcium–peptide chelate, and organic acid-bound calcium in the LA group were all almost twice as high as that in the HY group. Since LA fermentation produces more lactic acid [28], most of the organic acid-bound calcium may be in the form of calcium lactate in the LA group.

### 3.3. Particle Size and Morphology of Bone Powders

**Figure 3:**
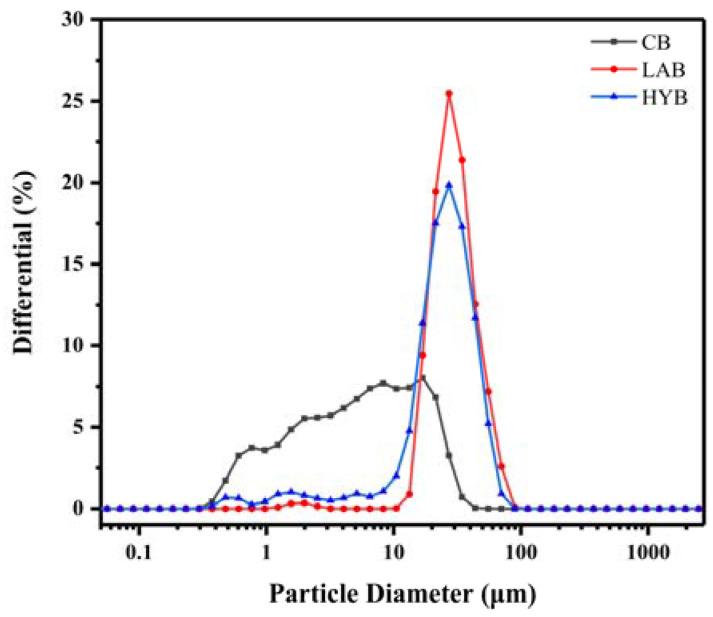
Particle size distribution of bone powders.

Table 2 and Figure 3 demonstrate the mean particle size and particle size distribution (PSD) of bone powders under the three methods of treatment. CB had the smallest mean particle size (4.04 μm), while HYB (21.495 μm) was larger than LAB (24.44 μm). CB had a wide range of particle sizes, with roughly 73.70% of the particles in the 0.4–10 μm range, and about 25.55% and 0.75% of the particles in the 10–30 and 30–50 μm ranges, respectively. It was noteworthy that fermentation enhanced the particle size and widened the PSD, as the particle sizes of LAB in the range of 10 to 35 µm accounted for 76.65% of the total, and the particle sizes in the range of 40–70 μm accounted for 9.90%. HYB exhibited not only the largest particle size, but also the widest PSD centered at 8–55 μm. 

**Figure 4:**
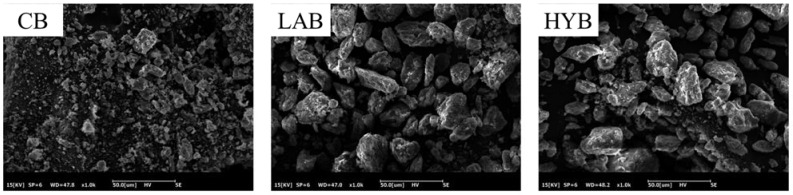
SEM images of bone powders (×500).

Figure 4 demonstrates the microstructures of CB, LAB, and HYB samples. All samples generally showed some degree of stacking, which may be associated with protein clumps adhered to the surface of the bone powders. CB particles had an irregular morphology, whereas the particles of LAB and HYB were more uniform. The particle size of HYB and LAB are larger than that of CB, which was consistent with the results of the particle size determination. The results showed that the microorganisms produced organic acids leading to the swollen collagen fibers and increased bone powder particle size.

### 3.4. Specific Surface Area and Pore Size Analysis of Bone Powders

**Figure 5:**
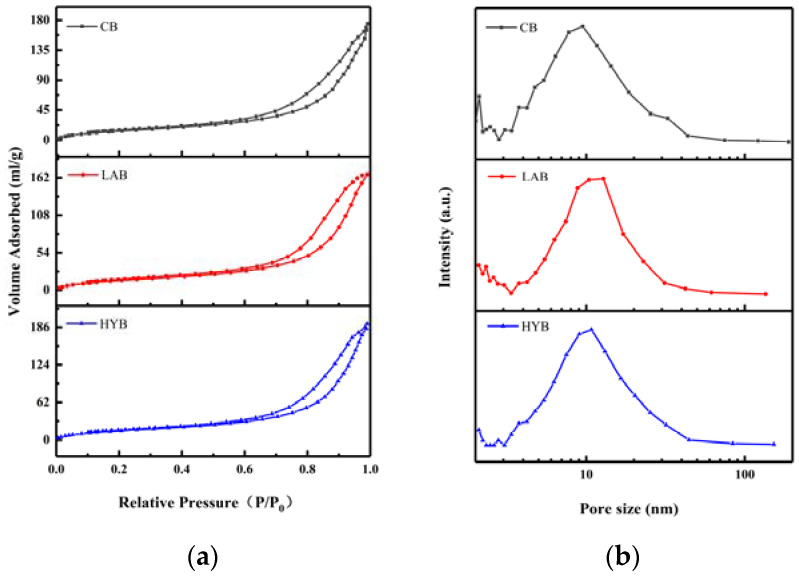
N_2_ adsorption–desorption isotherms (**a**) and pore size distribution (**b**) of fermented bone powders.

Figure 5a shows the N_2_ adsorption–desorption isotherms of different fermented bone powders, which exhibit the characteristics isotherms with H2 hysteresis loops of bone powders, suggesting the appearance of ink bottle pores with narrow entrances and large cavities. As shown in Figure 5b, micropores (< 2 nm) and mesopores (2–50 nm) coexisted in all samples, and the pore sizes are mainly concentrated between 9 and 20 nm. BET surface areas and average pore sizes of the samples are shown in Table 3. From the obtained BET data, it was evident that LAB and HYB witnessed a decrease of the specific surface area and an increase of the size of the pores compared to CB. The specific surface area of CB, LAB, and HYB particles were 201.03 m^2^/g, 56.37 m^2^/g, and 59.69 m^2^/g, respectively. The average pore sizes of CB, HYB, and LAB particles were determined to be about 12 nm, 18.3 nm, and 14.1 nm, respectively. The pore size of the bone powders increased significantly after fermentation, which might be due to the release of calcium resulting in the opening of the pore structure. The results were consistent with particle size and SEM images in Section 3.3.

### 3.5. X-ray Diffraction Analysis

**Figure 6:**
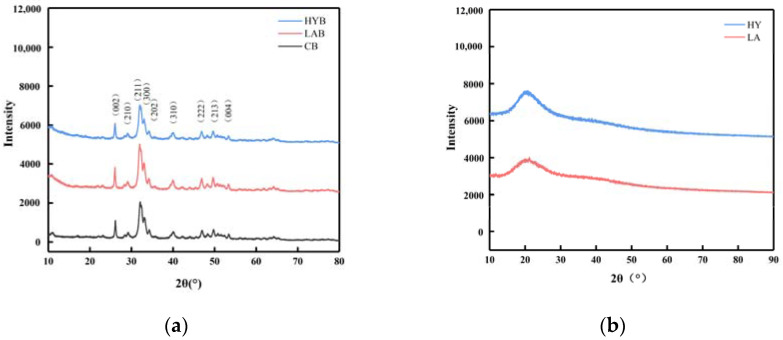
XRD patterns of bone powders (**a**) and calcium compounds in the fermentation broth (**b**).

Figure 6a showed the XRD patterns of the CB, LAB, and HYB samples. The diffraction patterns of the three samples were similar, which confirmed that they all had a hydroxyapatite (HAP) structure and a hexagonal crystal system (JCPDS Card No. 09-0432). Different from stoichiometric HAP, bone mineral is structurally disordered and nonstoichiometric in combinatorial position due to the presence of a large number of anionic and cationic (e.g., Na^+^, Mg^2+^) species, as well as the presence of ionic vacancies in the lattice [29]. CO_3_^2−^ ions, whose weight proportion can reach up to 5–9% in bone mineral, can occupy the PO_4_^3−^ (called B-type substitution) and/or OH^−^ (A-type substitution) sites within the hydroxyapatite’s crystal lattice [30]. The content and position of the carbonate ions in the crystalline structure has been reported to alter the unit cell parameters [12]. The unit cell parameters of HAP in CB, LAB, and HYB samples (Table 4) exhibited slightly different characteristics in comparison to the reported HAP, which could be due to the replacement of different types of carbonates. It was apparent that the parameter values of the three samples were different from those of HAP, type A, and type B CHAP, indicating the presence of all three types of apatite coexisting. As a result, it could be concluded that all samples displayed both types of carbonate substitutions.

Compared with CB, the increased intensity of the diffraction peaks in the LAB indicated that the crystallinity of the LAB was improved, while the weakened intensity of the diffraction peaks in the HYB revealed a decreased crystallinity. The organic acids produced in the fermentation process of LA group could react with the nonstoichiometric HAP and CHAP and collapse the disordered structure more easily, leaving mostly stoichiometric HAP with good crystallinity. However, for HY group, CO_2_ was produced during fermentation, which is easily soluble in water to form carbonate ions and enter into the crystalline structure of HAP to replace the OH^−^ or PO_4_^3−^ ions, leading to a decrease in the crystallinity of HYB.

On the basis of the above X-ray diffraction patterns and unit cell parameter analyses, although the three samples were processed by different methods, they verified similarities in phase composition and crystal structure, which included low crystallinity and a quantity of carbonates probably due to the acid produced by *Lactobacillus acidophilus* and yeast. The three samples were studied in further detail by FT-IR spectroscopy.

### 3.6. Fourier Transform Infrared Spectroscopy

**Figure 7:**
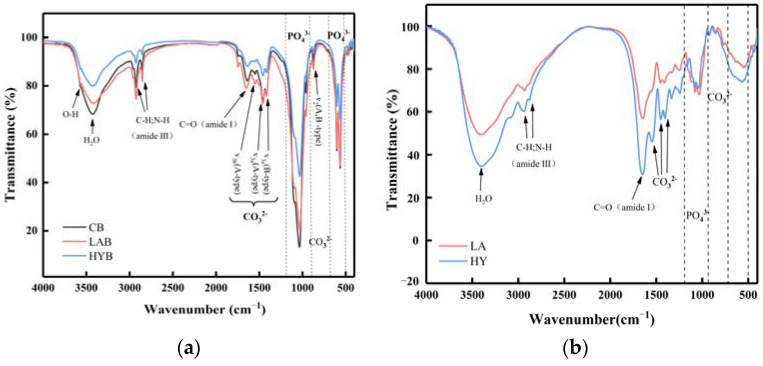
FT-IR spectra of bone powders (**a**) and compounds in the fermentation broth (**b**).

The infrared spectra of the three bone powder samples in Figure 7a were in similarity to those observed in hydroxyapatite minerals, which supported the results observed by XRD. The FTIR spectra of all the samples showed characteristic absorption bands for phosphate, hydroxyl, and carbonate, indicating that the inorganic matter mainly exists in the form of phosphate and carbonate in the samples [31].

The band assigned to the bending C=O (amide I) at 1650 cm^−1^ and the sharp bands observed at 2800–3000 cm^−1^ indicated the presence of aliphatic C-H and N-H stretching vibrations (amide III) characteristic of decomposed collagen [32]. In the spectrum, the amide I band of collagen was strong, while the displaced amide III band was weakened. Both of these spectral changes indicated a decrease in hydrogen bonding [33,34]. As shown in Figure 7a, the absorption peak located at the wavelength of 3567 cm^−1^ is assigned to hydroxyl. It was apparent from the intensity of the peaks that the hydroxyl content was in the following order: CB > LAB > HYB. The lower content of hydroxyl in LAB and HYB was probably due to the acids produced in the fermentation process, which reacted easily with the OH^−^ in HAP, thus indicating that the acidification promoted the disintegration and degradation of collagen fibers. HYB possessed the least content of hydroxyl, attributed to the substitution of hydroxyl by carbonate ions produced in the fermentation process.

The absorption peak of PO_4_^3−^ contained two regions. In the first region, the peak at 962 cm^−1^ and the peak at 1034 cm^−1^ were associated with *v1* and *v3* stretching modes, respectively. The second region had two bands at 603 and 564 cm^−1^ for the phosphate ions, which were due to the *v4* bending vibrations [35]. From the absorption peaks of the phosphate ions in Figure 7a, it could be observed that the content of PO_4_^3−^ followed in the order of CB > LAB > HYB. The content of PO_4_^3−^ was significantly decreased in LAB and HYB compared with CB, which was attributed to the decomposition of HAP from acid production by microbial fermentation and released PO_4_^3−^. Since the active dry yellow wine yeast produced more carbonate ions that easily replaced the PO_4_^3−^ during the fermentation, the PO_4_^3−^ content in HYB was less than LAB.

As shown in Figure 7a, all the samples exhibited two notable bands at 874 cm^−1^ and within the range of 1400 to 1600 cm^−1^, which belonged to the bending vibration out of the plane (*v2*) and the stretching vibration asymmetrically (*v3*) of carbonate ions, respectively. The peak appeared at 720 cm^−1^, corresponding to the *v*4 band of carbonate ions weak in CB and LAB, and not observed in HYB. This result was similar with that in the reference [36], in which the last peak of carbonate ions appeared at 720 cm^−1^, and usually displayed a relatively low intensity that is rarely observed. As notably observed in Figure 7b, HY group possessed higher carbonate ions content in the calcium compounds of fermentation broth compared to LA group, caused by the yield of CO_2_ through the fermentation process of active dry yellow wine yeast.

### 3.7. Analysis of Calcium–Phosphorus Ratio in Bone Powders

The major minerals in HAP (Ca_10_(PO_4_)_3_(OH)_2_) are calcium and phosphorus. The de termination of the calcium and phosphorus contents of ferment-treated bone powders could provide more direct evidence for the release of calcium from bone powders. The contents of calcium and phosphorus in CB were 609.38 mg/g and 342.91 mg/g, respectively, with the Ca/P molar ratio of 1.777 (Table 5). The ratio of calcium to phosphorous of all three samples was inconsistent with that in the stoichiometric Ca_10_(PO_4_)_3_(OH)_2_ (1.67), which was probably due to the presence of carbonate ions substituted HAP. Compared with CB, the ratio of calcium to phosphorous of LAB and HYB increased to 1.820 and 1.861, respectively. The decrease in calcium content in bone powders and the increase in calcium content in the fermentation broth suggested that fermentation can disrupt the structure of bone powders through acid production, and can cause calcium release from HAP.

### 3.8. Thermogravimetric Analysis

**Figure 8:**
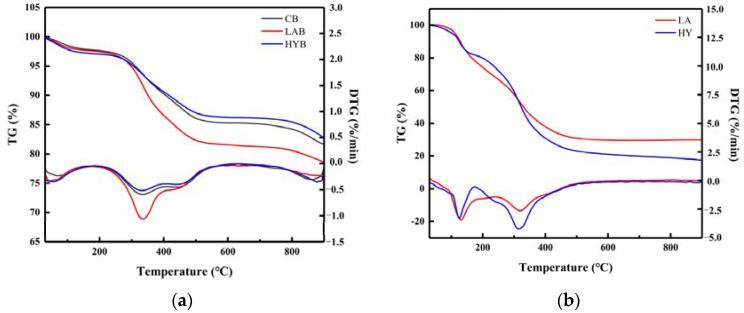
TG/DTG curves of bone powders (**a**) and compounds in the fermentation broth (**b**).

Figure 8a shows the TG and DTG curves of the three bone powder samples in the range of 20 °C to 900 °C. The weight loss of the three samples at a temperature of approximately 100 °C was attributed to the evaporation of water [37]. When the temperature was increased from 200 to 550 °C, the weight loss of the HYB was minimal in Table 6. In comparison, mass loss remained significant in the other two samples, which could be attributed to the decomposition of residual bone organic components, mainly collagen [38]. From the DTG curves, it can be seen that the decomposition rate of organic matter in LAB was faster than that in HYB, probably due to the swelling and decomposition of the collagen induced by higher acid production in the fermentation process of LA group. The third slope between 720 and 900 °C was determined to be the dehydroxylation and decomposition of hydroxyapatite in the bone powders. As shown in Table 5, the estimated amounts of weight loss in the range of 580 to 900 °C were about 3.64, 3.10, and 3.49% for CB, LAB, and HYB, respectively.

Figure 8b shows the thermal decomposition (TG and DTG) curves of compounds in the LA and HY fermentation broths in the temperature range of 30–900 °C. There were two main stages of mass loss in the samples. The first weight loss happened between 30 and 150 °C, attributed to the evaporation of free water molecules and bound water from samples. The second slope between 200 °C and 400 °C showed a continuous decrease in the mass of the samples, mainly caused by the decomposition of the calcium lactate and the organic matter (glucose, peptone) in the samples. In Table 7, the total weight loss in the second step of LA and HY was 36.28% and 49.03%, respectively.

## 4. Discussion

**Figure 9:**
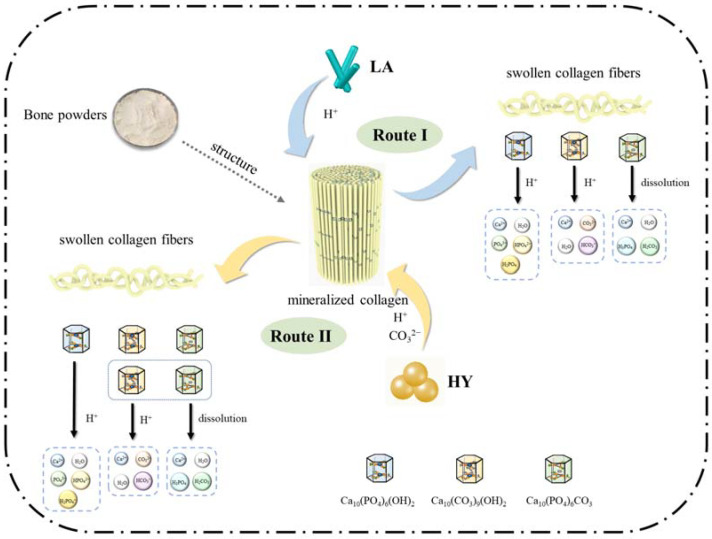
Proposed mechanism of calcium changes in bone powders after fermentation.

On the basis of the above results and discussions, the schematic proposal is illustrated in Figure 9. The mechanism in the fermentation process of bone powders by LA and HY was similar, during which the microorganisms produced organic acids leading to the swollen collagen fibers and the changes of hydroxyapatite crystals in the bone. However, the details were different and classified into Route I and Route II in Figure 9. In Route I, the *Lactobacillus acidophilus* produced a large amount of H^+^, which reacted with hydroxyl in HAP and B-type CHAP (Ca_10_(CO_3_)_9_(OH)_2_) and disrupted the structure of crystals. In the meantime, the solubility of A-type CHAP (Ca_10_(PO_4_)_6_CO_3_) could increase under acidic conditions, leading to the dissolution despite the fact that A-type CHAP is unable to react with H^+^ due to the lack of hydroxyl. However, in Route II, the active dry yellow wine yeast produced CO_2_ during fermentation, which formed CO_3_^2−^ under acidic conditions, leading to the higher content of A-type and B-type CHAP.

## 5. Conclusions

In pursuit of an in-depth exploration into biocalcium supplements and the enhanced utilization efficiency of animal bones, this study delved into the fermentation processes applied to cattle bone powders and the associated mechanism. This investigation focused on the impact of two distinct microbial agents, namely *Lactobacillus acidophilus* and active dry yellow wine yeast, on calcium release, particle size, microstructure, and chemical composition of cattle bone powders. Throughout the fermentation process, microorganisms, notably *Lactobacillus acidophilus* and yeast, generated a substantial quantity of H^+^, disrupting the structure of bones, characterized by swollen collagen fibers, the release of Ca^2+^, an increase of particle size, and modifications in surface bone calcium composition (higher content of A-type and B-type CHAP). Analysis of the fermentation broths revealed that calcium manifested primarily in the forms of free calcium, organic acid-bound calcium, and peptide-bound calcium. Notably, *Lactobacillus acidophilus* exhibited superior acid production capacity and enhanced calcium release efficacy compared to the yeast, whereas yeast fermentation resulted in CO_2_ production, which formed CO_3_^2−^ under acidic conditions, contributing to elevated levels of A-type and B-type CHAP. These findings not only pave the way for the fermentation-driven production of calcium supplements, but also establish a robust foundation for the exploration of potential calcium supplements characterized by favorable bioavailability.

## Figures and Tables

**Table 1:** Content of different forms of calcium in bone powder fermentation broths.

Group	Total Calcium (mg/L)	Free Calcium Ion (mg/L)	Calcium–Peptide Chelate (mg/L)	Organic Acid-Bound Calcium (mg/L)
Control	124.9 ± 6.035 ^c^	106.51 ± 0.323 ^c^	13.32 ± 2.520 ^c^	5.07 ± 7.671 ^c^
LA	1617.47 ± 17.771 ^a^	827.34 ± 14.149 ^a^	62.9 ± 7.065 ^a^	727.23 ± 8.681 ^a^
HY	646.57 ± 15.489 ^b^	316.02 ± 6.334 ^b^	41.3 ± 0.821 ^b^	289.25 ± 11.645 ^b^

The different letters indicate a significant difference (*p* < 0.05).

**Table 2:** Mean particle size of fermented bone powders.

Group	Mean Particle Size (μm)
CB	4.032 ± 0.315 ^b^
LAB	21.495 ± 1.097 ^a^
HYB	24.444 ± 1.391 ^a^

The different letters indicate a significant difference (*p* < 0.05).

**Table 3:** Specific surface area and mean pore size of the samples.

Sample	Specific Surface Area(m^2^/g)	Pore Size(nm)
CB	201.03	12.00
LAB	56.37	18.30
HYB	59.69	19.85

**Table 4:** The unit cell parameters of CB, LAB, HYB, HAP, and carbonated (A-and B-types) CHAP.

	CB	LAB	HYB	HAP	A-Type CHAP	B-Type CHAP
Space group	P6_3_/m	P6_3_/m	P6_3_/m	P6_3_/m	Pb	P6_3_/m
a (Å)	9.352	9.4166	9.4166	9.421	9.527	9.386
b (Å)	-	-	-		-	-
c (Å)	6.882	6.8475	6.8475	6.88	6.875	6.901

**Table 5:** Calcium and phosphorus content of bone powders in three different treatment groups.

Samples	Ca (mg/g)	P (mg/g)	Ca/P
CB	609.38 ± 5.260 ^a^	342.91 ± 4.237 ^a^	1.777 ± 0.012 ^c^
LAB	538.70 ± 4.107 ^c^	295.92 ± 3.397 ^b^	1.820 ± 0.015 ^b^
HYB	561.85 ± 6.803 ^b^	301.75 ± 4.937 ^b^	1.861 ± 0.014 ^a^

The different letters indicate a significant difference (*p* < 0.05).

**Table 6:** Characteristic parameters of thermogravimetric analysis of bone powders.

Sample	Mass Loss (%)		
	30~150 °C	200~550 °C	580~900 °C
CB	2.08%	11.55%	3.64%
LAB	2.28%	15.13%	3.10%
HYB	2.73%	10.04%	3.49%

**Table 7:** Characteristic parameters of thermogravimetric analysis of compounds in the fermentation broth.

Sample	Mass Loss (%)	
	30~150 °C	200~400 °C
LA	16.72%	36.28%
HY	16.12%	49.03%

## Data Availability

Data is contained within the article.
